# The instability of the BTB-KELCH protein Gigaxonin causes Giant Axonal Neuropathy and constitutes a new penetrant and specific diagnostic test

**DOI:** 10.1186/2051-5960-2-47

**Published:** 2014-04-24

**Authors:** Alexia Boizot, Yasmina Talmat-Amar, Deborah Morrogh, Nancy L Kuntz, Cecile Halbert, Brigitte Chabrol, Henry Houlden, Tanya Stojkovic, Brenda A Schulman, Bernd Rautenstrauss, Pascale Bomont

**Affiliations:** 1 Atip-Avenir team, Inserm U1051, Institut des Neurosciences de Montpellier, Montpellier, France; 2 Université de Montpellier1&2, Montpellier, France; 3 NE Thames Regional Genetics Service, Great Ormond Street Hospital, York House Queen Square, London, UK; 4 Northwestern University Feinberg School of Medicine, Chicago, USA; 5 Service de Neurologie Pédiatrique, Hôpital d’Enfants, CHU Timone, Marseille, France; 6 Department of Molecular Neuroscience and The MRC centre for Neuromuscular Diseases, Institute of Neurology, Queen Square, London, UK; 7 AP-HP, G-H Pitié-Salpêtrière, Institut de Myologie, Paris, France; 8 Howard Hughes Medical Institute, Department of Structural Biology, St. Jude Children’s Research Hospital, Memphis, Tennessee, USA; 9 Medizinisch Genetisches Zentrum Bayerstrasse, München, Germany; 10 Ludwig-Maximilians-University Friedrich-Baur-Institut, München, Germany

**Keywords:** CMT2, GAN, Diagnosis, Gigaxonin, E3 ligase, Modelization, Instability

## Abstract

**Background:**

The BTB-KELCH protein Gigaxonin plays key roles in sustaining neuron survival and cytoskeleton architecture. Indeed, recessive mutations in the Gigaxonin-encoding gene cause Giant Axonal Neuropathy (GAN), a severe neurodegenerative disorder characterized by a wide disorganization of the Intermediate Filament network. Growing evidences suggest that GAN is a continuum with the peripheral neuropathy Charcot-Marie-Tooth diseases type 2 (CMT2). Sharing similar sensory-motor alterations and aggregation of Neurofilaments, few reports have revealed that GAN and some CMT2 forms can be misdiagnosed on clinical and histopathological examination. The goal of this study is to propose a new differential diagnostic test for GAN/CMT2. Moreover, we aim at identifying the mechanisms causing the loss-of-function of Gigaxonin, which has been proposed to bind CUL3 and substrates as part of an E3 ligase complex.

**Results:**

We establish that determining Gigaxonin level constitutes a very valuable diagnostic test in discriminating new GAN cases from clinically related inherited neuropathies. Indeed, in a set of seven new families presenting a neuropathy resembling GAN/CMT2, only five exhibiting a reduced Gigaxonin abundance have been subsequently genetically linked to GAN. Generating the homology modeling of Gigaxonin, we suggest that disease mutations would lead to a range of defects in Gigaxonin stability, impairing its homodimerization, BTB or KELCH domain folding, or CUL3 and substrate binding. We further demonstrate that regardless of the mutations or the severity of the disease, Gigaxonin abundance is severely reduced in all GAN patients due to both mRNA and protein instability mechanisms.

**Conclusions:**

In this study, we developed a new penetrant and specific test to diagnose GAN among a set of individuals exhibiting CMT2 of unknown etiology to suggest that the prevalence of GAN is probably under-evaluated among peripheral neuropathies. We propose to use this new test in concert with the clinical examination and prior to the systematic screening of GAN mutations that has shown strong limitations for large deletions. Combining the generation of the structural modeling of Gigaxonin to an analysis of Gigaxonin transcripts and proteins in patients, we provide the first evidences of the instability of this E3 ligase adaptor in disease.

## Introduction

Gigaxonin is a BTB-KELCH protein that plays a central role in sustaining neuron integrity and cytoskeleton architecture. Indeed, recessive mutations in the Gigaxonin-encoding gene are responsible for a devastating neurodegenerative disorder in human, called Giant Axonal Neuropathy (GAN [MIM 256850]) [1], that leads to a wide deterioration of the nervous system and provokes a massive disorganization of the Intermediate Filament (IF) cytoskeleton.

Diagnosed early in infancy, the disease first touches the peripheral nervous system, altering both the motor and sensory tracts in teens, and closely resembles to the most common inherited peripheral neuropathy called Charcot-Marie-Tooth (CMT) diseases. Thus, patients exhibit weakness and severe wasting of the four limbs predominating in distal segments, sensory and motor loss, and reduced deep tendon reflexes. Symptoms evolve towards areflexia, loss of the deep and superficial sensitivity and loss of ambulation. Subsequently, the disease targets the central nervous system, leading to a wide range of symptoms encompassing ataxia, nystagmus, dysarthria and intellectual disability [2,3]. Fatal in young adults, GAN is a progressive neurodegenerative disorder of axonal type. Although few milder cases of the disease with later onset, absence of central nervous system impairment, or longer survival have been described, GAN invariably causes the massive collapse of IFs in a variety of tissues, including Neurofilaments (NFs) in distended or “giant” axons in nerve biopsy [2,3]. Up to recently, NF aggregation in giant axons constituted a powerful histological test towards the diagnosis of GAN, which is now compromised by similar histopathological findings in several forms of CMTs [4,5].

While NF aggregation has been reported in many neurodegenerative disorders, including Alzheimer’s, Parkinson’s diseases and Amyotrophic Lateral Sclerosis, possibly as a results of neuron injury, the disorganization of all classes of IFs is unique to GAN and supports a crucial role of Gigaxonin in sustaining cytoskeleton architecture [6]. In addition to neuronal IF defects, GAN patients display aggregation of GFAP, desmin, keratin and vimentin. In GAN patient-derived primary fibroblasts, vimentin aggregation has been shown to be reversible, conditional, and independent of microtubule overall stability [7,8]. The central role of Gigaxonin in regulating IFs has been confirmed in GAN mouse models, although these only exhibit mild motor and sensory deficits with no signs of robust neurodegeneration [9,10]. Indeed, Gigaxonin-depleted mice display a massive aggregation, spatial disorganization and increased abundance of several IF proteins throughout the central and peripheral nervous system [9,10].

How this low abundance BTB-KELCH protein [8], preferentially expressed throughout the nervous system and during development [10], controls neuron survival and IF architecture remains unknown. One plausible hypothesis is that this would be mediated by the Ubiquitin Proteasome System (UPS) pathway. Indeed, BTB-containing proteins, including Gigaxonin have been identified as the substrate adaptors of Cul3-E3 ubiquitin ligases, mediating the addition of ubiquitin chains onto their targets prior to their degradation by the proteaseome [11-13]. Interacting with the E3 ligase complex to its N-terminal BTB domain, Gigaxonin is thought to promote the Ubiquitin tagging of the substrates through interaction with its C-terminal KELCH domain. Although Gigaxonin has been shown in cells to participate in regulating the abundance of three regulators of microtubules, the E3 ligase activity of Gigaxonin remains to be fully established. A first step towards this goal has been recently reached, in a study revealing that the overexpression of Gigaxonin induces the degradation of several IF proteins, including vimentin in primary fibroblasts and that this clearance involves the proteasome [14].

To better understand the disease mechanisms in GAN and provide a specific diagnostic tool able to discriminate GAN from closely related CMTs, we combine here a study on Gigaxonin abundance and stability in disease and a structural modeling of Gigaxonin to a prognostic study on new patients presenting a sensorimotor neuropathy of unknown etiology. We establish that in GAN patients, mutant Gigaxonin levels are greatly reduced in abundance. The quantification of Gigaxonin mRNA reveals nonsense mRNA decay as one of the disease mechanisms in GAN. In addition, the modelization of Gigaxonin structure allows us to map GAN mutations and predict a general destabilization of disease-associated mutants, which is further confirmed by reduced half-lives of mutant Gigaxonins. Finally, we establish that our immunodetection of Gigaxonin constitutes a robust, penetrant and specific new diagnostic test for GAN, circumventing the limitations of gene sequencing and the clinical and histopathological overlap between GAN and the frequent forms of axonal CMTs.

## Materials and methods

### Preparation of lymphoblast cell lines from patients

Blood samples were collected from patients with written informed consents and under the agreement n° DC-2010-1191 of the Bioethic comittee of the Ministère de l’Enseignement Supérieur et de la Recherche. Numbering of previously reported GAN patients matches publications [1,15] for F1-F18 families, and [16] for family F25. New families included in the study (F23, 24, 26–30) were addressed to banque d’ADN et de Cellules de Généthon (Evry, France) for the generation of immortalized cell lines.

### Immunoblotting

Cell lines, expanded in RPMI 20% FBS, 1% P/S and 2 mM Glutamine (Invitrogen), were lysed and processed for western blotting as previously described in [8]. Primary antibodies are: Gig A (1:150) [8], DM1α-tubulin from Merck Millipore n°CP06 (1:10000); GAPDH from Ambion n°4300 (1:4000). Quantification was performed on 3–5 independant experiments with Image Lab (Biorad) after normalization with Tubulin or GAPDH. Statistical analysis was performed using Prism GraphPad.

### Genetic analysis

All coding exons and flanking intron sequences of the *GAN* gene were Sanger sequenced using primers described in [1], the Big Dye Terminator v3.1 sequencing kit and analyzed on an ABI3130 Genetic Analyzer (Applied Biosystems, USA). The reference sequence used for GAN was the NM_022041.2. Variants were compared to the known public databases (dbSNP, 1000 genomes), the Inherited Peripheral Neuropathies Mutation Database (IPNMDB; http://www.molgen.ua.ac.be/CMTmutations/default.cfm) and Human Gene Mutation Database (HGMD) to exclude polymporphisms in the normal population.

Genomic rearrangement on the *GAN* gene was analyzed on proband and family genomic DNA by High-resolution custom NimbleGen 135 k CGH microarray (probe spacing of 75 bp for exons and 200 bp for the introns) versus reference DNA (Kreatech, Amsterdam, The Nederlands). The array also included a genomic backbone probe set with an average probe spacing of 30 kb. DNA samples were labeled (test with Cy3 and reference with Cy5) and co-hybridised to the custom microarray in accordance with the manufacturer’s instructions (NimbleGen Arrays User’s Guide: CGH and CGH/LOH Arrays v9.1, Roche NimbleGen, Madison, WI USA). The microarray was washed and then scanned on an Axon GenePix 4400A Scanner using GenePix Pro 7 software (Molecular Devices, Sunnyvale, CA, USA). Raw data was normalized, LOESS correction applied and the data ratios calculated using DEVA v1.01 Software (Roche NimbleGen). The normalized data was processed using Infoquant Fusion v6.0 software (Infoquant, London, UK) with analysis call settings of 3 consecutive probes +/- 0.4 Cy3/Cy5 ratios. The arrays were used on affected and unaffected family individuals as well as normal controls.

### Quantitative RT-PCR (qRT-PCR)

Total RNA from patients and control cell lines was isolated using the RNeasy MicroKit (Qiagen) according to the supplier’s recommendations. For each sample, 1 μg of RNA was used for reverse transcription with oligodT primers and SuperScriptIII (Invitrogen). SYBR green quantitative real-time PCR was performed with LightCycler 480 SYBR Green I Master (Roche) in a two-step cycling protocol on 100 ng of cDNA, using GAN-exon9-11 specific primers (GGGTAGCGAGATGGTAACTTG and CGGATGGAAGGAGTGGTTTAG) and HPRT1 quantitect primers (Qiagen). Carrying a deletion in the GAN exons 10 to 11, F24 mRNA level was determined by another set of primers, *i.e.* GAN-exon4-5 (QT00018774, Qiagen), together with appropriate positive and negative controls. The relative abundance of the patient’s mRNA was expressed as the fold change to the controls mRNAs. Fold changes were measured as the ratio of the ΔΔCT of each patient to the ΔΔCT of the controls after normalization with HPRT1. Three independent RT-PCR were performed in triplicate for each sample. Accordingly to the manufacturer’s instructions, only fold changes exceeding a value of 2 are considered significantly different.

### Statistical analysis

The student’s *t*-Test was used to determine statistical significance. Error bars represent standard deviation and p values are reported in the Figure legends.

### Structural modeling

The structural model of the BTB-BACK domain Gigaxonin bound to the N-terminal domain of CUL3 was generated as follows. Residues 8–128 were used from the crystal structure of the BTB domain of Gigaxonin, which also included a partial model for the BACK domain (3HVE.pdb, [17]). A complete model for the BACK domain (residues 129–256) was obtained from the Phyre homology modeling server (http://www.sbg.bio.ic.ac.uk/phyre2/) based on the crystal structure of human KLHL11 (3I3N.pdb) [18]. The BTB domain from Gigaxonin was superimposed on that from SPOP in complex with the N-terminal domain from CUL3 (4EOZ.pdb) to add this portion of CUL3 to the model [19]. The KELCH domain of Gigaxonin (residues 273–577) was modeled with the crystal structure of the KELCH domain of the BTB-BACK-KELCH protein Keap1, using the Phyre server with 100% confidence for the fold and based on 1X2R.pdb (27% sequence identity) [20,21].

### Pulse chase assay

COS cells were transfected by plasmids expressing human wild type or mutated Gigaxonin tagged with a N-terminal Flag sequence, using Fugene 6 transfection (Promega). R138H, L309R, R477X and R15S correspond to mutations of families F13, F1, F16 and F2, respectively. WT, N-ter and C-ter correspond to the Full-length Gigaxonin, the BTB domain (residues 1 to 223) and the KELCH domain (residues 141 to 597), respectively. 24 hours post transfection, cells were washed twice with PBS, incubated in methionine- and cysteine-free DMEM (Sigma) supplemented with 10% FBS and 1% P/S for 1 h before labeling with 100 μCi of S^35^-methionine/cysteine (Perkin Elmer) for 45 min. Cells were subsequently washed twice with PBS and incubated with normal DMEM medium, containing serum and antibiotics. Cells were washed with PBS and collected by centrifugation at the beginning of the chase period (0 h) and at 2, 4, 6, 9, 24 hours. Protein extracts were lysed as previously described for lymphoblast cell lines and immunoprecipitation was performed on 60 μg of total proteins using anti Flag antibody (Flag-M2, sigma) and G-protein (Dynabeads, Fisher scientific). Immunoprecipates were recovered in Laemmi buffer and loaded on two identical gels for autoradiography and immunoblotting (for internal control). Because immunoblotting of Gigaxonin (using either the Flag-M2 or the mouse Gigaxonin antibody (GigA, [8]) hampered the detection of several mutants/truncated Gigaxonin due to cross reaction with mouse IgG, we used the supernatents recovered after the incubation of the Gigaxonin-Antibody complex with the G-protein as the best internal control, using tubulin (DM1α, 1:10000). Three independent labeling-immunoprecipitation experiments were performed per condition. All quantifications were performed with Image Lab (Biorad) after normalization with Tubulin. Half-lives were determined with Prism (Non linear regression).

## Results

### Decreased abundance of GAN-linked Gigaxonin

**Figure 1:**
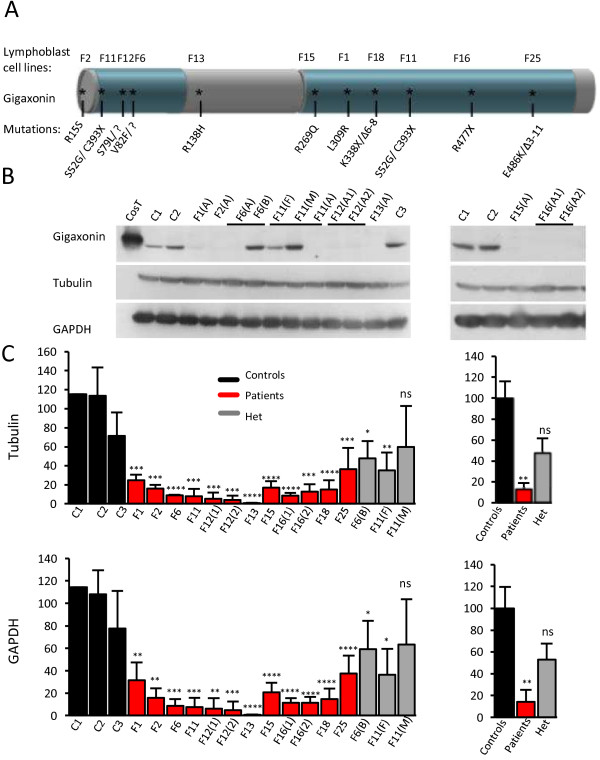
**Decreased abundance of disease-associated Gigaxonin. A** Schematic representation of Gigaxonin and the corresponding known mutations in GAN patients. The N-terminal BTB and C-terminal KELCH domains are represented in blue. Lymphoblast cell lines derived from GAN patients are numbered F1-F25 and their respective mutations are mapped on Gigaxonin. All patients are severely affected by the disease with the exception of patients F2 and F13, who are mild cases reported previously. **B** Abundance of Gigaxonin, as revealed by immunoblotting using the GigA antibody [8]. Cost and c1-c3 correspond to ectopic Flag-tagged Gigaxonin expressed in COS cells and to unrelated control individuals, respectively. (A), (B), (F) and (M) stand for Affected, non-affected Brother, Father and Mother, respectively. A1 and A2 are two affected children from the same family. Please note that immunoblottings of patients F18 and F25 are shown in Figure 2A. **C** Quantification of Gigaxonin in GAN patients and their relatives. Left: Percentage of Gigaxonin for each individual in comparison to wild type Gigaxonin, as the average of 3–5 independent experiments, after normalization with tubulin and GAPDH. Right: Mean abundance of Gigaxonin in patients and heterozygous individuals, as measured by the percentage in comparison to wild type Gigaxonin. (T-test, *, p < 0,05; **, p < 0,01, ***, p < 0,001 and ***, p < 0,0001; error bars represent standard deviation).

Inherited through a recessive mode of inheritance, GAN is suspected to result from a Gigaxonin loss of function mechanism. We previously showed a dramatic decrease in the abundance of mutant Gigaxonin in few GAN patients using an immunodetection method on immortalized lymphoblast cell lines [8]. To further confirm this finding on a larger group of patients and determine the levels of residual mutated Gigaxonin, we quantified the abundance of Gigaxonin in all GAN patients for which immortalized lymphoblast cell lines have been derived and mutations identified (Figure 1). Those include eight severely affected patients and two mild cases (R15S and R138H mutations, corresponding to family F2 and F13 in Figure 1A). Univocally, all mutated Gigaxonins, including severe but also mild cases were considerably less abundant than wild type proteins (Figure 1B). Thus, the levels of mutant Gigaxonin ranges from [0,73 to 36,6]% of the wild type counterparts, with a mean value of 13,1 ± 5,7 using normalization with tubulin, and a [0,6-37,6]% range with a mean value of 14,2 ± 10,9 with GAPDH (Figure 1C). In an attempt to determine whether heterozygous compounds, carrying one wild type Gigaxonin in addition to one mutated form may exhibit a dose effect in protein level, we quantified its abundance in patient’s relatives: unaffected brother of patient F6 and both parents of patient F11 (Figure 1B, C). Very interestingly, the level of Gigaxonin in heterozygous individuals ranges from [35,2-59,9] or [36,3-63,4]% of wild type Gigaxonin using tubulin or GAPDH normalizators, respectively. Whereas the mean values of 47,7 ± 14,2 or 53,0 ± 14,6 represent half of the abundance of healthy individual (carrying 100% of wild type proteins), this is not statistically significant, probably due to the intra-individual (as seen for mother of F11) and inter-individual variability between control samples. Therefore, we conclude that when mutated in both alleles, Gigaxonin is greatly reduced in abundance by 85,8% in average and by 99,3% in the most extreme case, but that additional control and heterozygous individuals should be tested to convincingly discriminate the latter from healthy individuals.

### Gigaxonin abundance discriminates GAN from closely related CMT diseases

To assess whether the determination of Gigaxonin abundance could contribute to diagnose GAN, we included in the study seven new patients presenting a sensorimotor axonal neuropathy resembling GAN/CMT2 with unknown genetic etiology (Table 1). Some patients have simultaneously been evaluated clinically [22].

**Table 1:** Phenotypic data of patients

	**F23**	**F24**	**F26**	**F27**	**F28**	**F29**	**F30**
**Country of origin**	**India**	**USA**	**USA**	**New Zealand**	**Northern Europe**	**Portugal**	**North Africa**
Consanguinity	-	-	na	-	-	Same village	1 st
Gender	F	F	M	M	F	F	F
Onset (year)	4	2,5	2	3	2	11	4
Present age	10	9	10	22	7	38	20*
Muscle weakness/tone	+	+	+	+	+	+	+
Reduced MNCV^a^	+	+	+	+	na	+	+
Loss of independence (year) or	8	3	3	10	na	-	na
Total loss of ambulation (year)	sa	sa	8	18	7	sa	8
Reduction of sensibility^b^	S, D	S, D	S, D	S, D	S, D	S, D	S, D
Areflexia (lower limbs)	+	+	+	+	-	+	+
Vision^c^	O, N	N	O, N	N	N	Normal	N
Dysarthria	+	+	+	-	+	-	+
Ataxia	+	+	+	-	+	-	+
Kinky hairs	+	+	+	-	+	-	+
Giant axon & NF aggregation	+	+	+	ni	ni	+	+
Suspicion of GAN	**Severe**	**Severe**	**Severe**	Mild	**Severe**	Mild	**Severe**
*GAN* mutation	c.724C > T	c.10-11del	c.146C > A	c.[971C > T]; [1391G > A]	-	-	C.994G > A
(Figure 2)	R242X		A49E	A324V/C464Y			G332R

*Deceased; na, not available; sa, still ambulant; ni, not investigated; -, absence.

1^st^ degree of consanguinity means that parents are first cousins.

^a^MNCV, motor nerve conduction velocity; ^b^S: Superficial sensitivity (light, touch temperature…); D: deep sensitivity (vibration, space…); ^c^O: optic atrophy; N: nystagmus.

The clinical presentation of patients F23-F27 is further detailed in Roth et al., [22].

Among the seven new patients, five of them, namely patients F23, F24, F26, F28 and F30 present an early (<4 years) onset progressive neuropathy indicative of the typical severe form of GAN. As revealed by the reduction of nerve conduction velocities, they exhibit an axonal motor and sensory neuropathy with muscle weakness/tone, areflexia that evolves to the loss of ambulation and of the deep and superficial sensitivity during childhood. All patients subsequently develop central nervous system impairment encompassing nystagmus, dysarthria and ataxia. Concomitant with these clinical signs, GAN has been shown to induce a wide aggregation of the cytoskeletal IF network both in and out-side the nervous system, and severely affected patients F23, F24, F26, F28 and F30 all exhibit altered keratins (kinky hair) and aggregating NFs in enlarged axons (Table 1).

Two additional patients (F27 and F29) present a neuropathy differing from the GAN typical form, with ± late onset, mild central nervous system impairment, extended survival but with the presence of giant axons and NF aggregation that may suggest a milder form of GAN or another related sensori-motor neuropathy called type 2 Charcot-Marie-tooth (CMT) disease. Indeed, we previously identified two moderate forms of the disease with very slow progression, no central nervous impairment and extended survival (F2 in the present study, carrying a R15S mutation), or with a late onset at 10 years of age, slow evolution with no central system involvement for patient F13 (with a R138H mutation).

**Figure 2:**
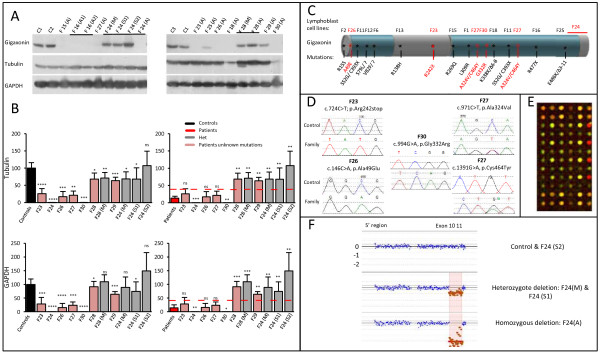
**Diminished levels of Gigaxonin corroborate with identification of mutations in the GAN locus. A** Immunodetection of Gigaxonin in new patient’s lymphoblast cell lines. (S1) and (S2) are unaffected sisters of patient F24. **B** Quantification of Gigaxonin in patients and their relatives, using Tubulin or GAPDH as internal controls. Individual level of Gigaxonin is compared with the range of wild type Gigaxonin (left panel) and mutated Gigaxonin in known GAN patients (as presented in Figure 1, right panel). The red lines correspond to the maximum individual mean value from patients. Please note that Gigaxonin abundance was so low (undetectable) for F24 and F30 that it was detected as significantly different from mutated Gigaxonin. N = 3-5 experiments. (T-test, *, p < 0,05; **, p < 0,01, ***, p < 0,001 and ***, p < 0,0001; error bars represent standard deviation). **C** Schematic representation of Gigaxonin and the mutations identified in known patients (black) and new patients (red). **D** Electropherograms representing the point mutations identified by systematic screening of the 11 exons of the *GAN* gene. **E** Illustration and **F** results of the CGH data, that revealed homozygous genomic deletion encompassing exons 10 and 11 in patient F24 and heterozygosity for sister 1*.*

The quantification of Gigaxonin in patients suspected of bearing a GAN severe (F23, F24, F26 and F30) and mild (F27) forms revealed a considerable diminution of abundance using both normalization methods and that is comprised in the range established for GAN patients with identified mutation (Figure 2A). Thus, Gigaxonin levels reach 25,7 ± 14,8% of the wild type Gigaxonin for patients F23; 16,9 ± 15,4% for patient F26; 21,3 ± 12,3% for patient F27 and no detectable Gigaxonin could be detected for patient F24 and F30 (Figure 2A, left panels). Gigaxonin levels were compared to the mean abundance of wild type Gigaxonins and mutated Gigaxonins in known GAN cases (Figure 2, left and right panels, respectively). This analysis showed that all patients have Gigaxonin levels that differ from wild type but not from mutated Gigaxonins (Figure 2B), suggesting that they are genetically linked to GAN. Testing relatives of patient F24 revealed that only the mother and sister S1 show a different abundance in comparison to control individuals, therefore suggesting that, as the mother, this sister may carry one mutated allele, whereas the other sister may not.

Surprisingly, patient F28 who shows a severe clinical presentation similar to GAN presents level of Gigaxonin that differ both from the range of normal Gigaxonin and mutated Gigaxonin (Figure 2B, left and right panels, respectively). As for the atypical patient F29, our quantification method revealed intermediate levels of Gigaxonin that could reflect an inter-individual variation of wild type Gigaxonin, as suggested earlier (Figure 1). This assumption was reinforced by the fact that the healthy mother of patient F28 expresses the same amount of Gigaxonin than her affected child: respectively 71,1 ± 16% versus 63,3 ± 17,5 using tubulin noralization and 109,7 ± 25,1% versus 91,5 ± 19,1 for GAPDH.

To determine whether the decreased abundance of Gigaxonin found in some patients can be corroborated by genetic alteration in the *GAN* gene, a systematic screening for point mutations and genomic rearrangement was performed in the GAN locus. This analysis revealed that all patients with reduced Gigaxonin level below the 37,6% maximum level established for known GAN cases (from Figure 1), carry a mutation in the *GAN* gene (Figure 2C). More specifically, Gigaxonin displays a premature stop codon in amino acid position 242 (R242X) on both alleles for patient F23; a large homozygous deletion encompassing exon 10 and 11 in patient F24, the exact deletion is from chr16: 81,402,224 to 81,411,392; a A49E missense mutation at homozygous state for patient F26, a compound heterozygous mutations A324V/C464Y for patient F27 and a homozygous G332R for patient F30 (Figure 2C-F). Conversely, when Gigaxonin levels were not compatible with our GAN known range (as for patient F28 and F29), neither point mutation nor chromosomal rearrangement could be revealed in the GAN locus. It is interesting to note that the CGH analysis revealed that Sister S1 of patient F24, suspected from her Gigaxonin level to be heterozygous carrier displays indeed this large deletion on one allele, whereas sister 2 with normal Gigaxonin level does not carry any deletion. Thus, our study provides evidences that abundance of Gigaxonin is not only univocally considerably diminished in all GAN patients reported so far, but that its quantification constitutes an essential tool to discriminate GAN from other hereditary polyneuropathies.

### Activation of non sense mediated mRNA decay in GAN

**Figure 3:**
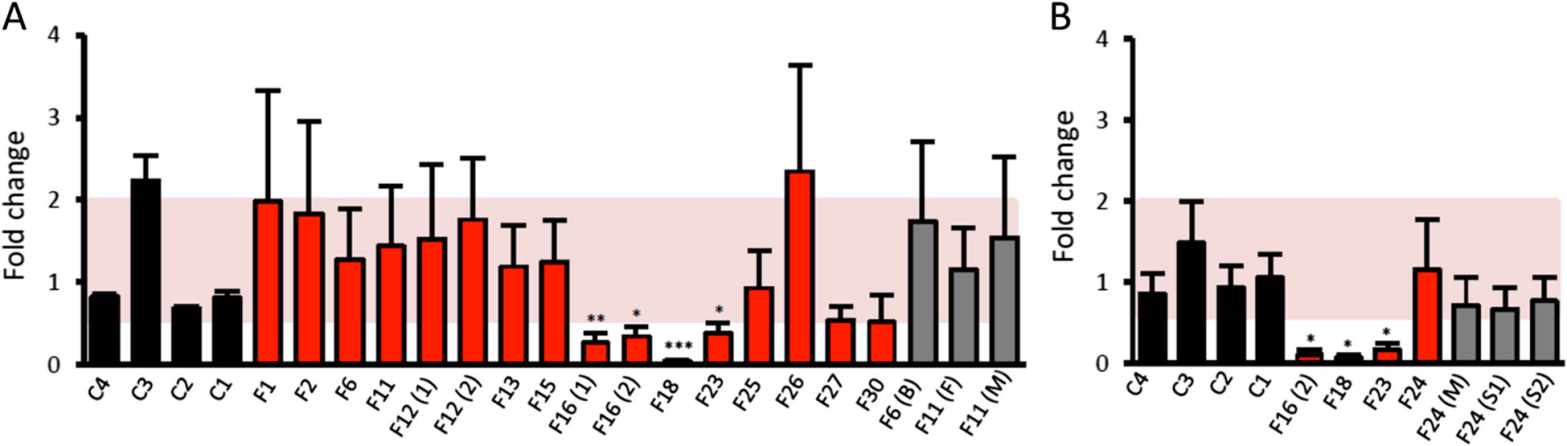
**Nonsense mediated mRNA decay in some GAN patients.** Gigaxonin mRNA levels of four control individuals (c1-4, in black), all GAN patients (in red) and their relatives (in grey) are measured using quantitative RT-PCR with GAN-exons9-11 **(A)** and GAN-exons4-5 **(B)**, and normalized using HPRT mRNA levels. Each mRNA level is expressed as the fold change to the mean value of the four control mRNAs. (B), (F) and (M) (S1 or S2) stand for non-affected Brother, Father, Mother, and sister, respectively N = 3 experiments. (T-test, *, p < 0,05; **, p < 0,01, ***, p < 0,001; error bars represent standard deviation SD. A 2-fold change is statistically different).

To determine whether the decreased abundance of GAN-linked Gigaxonins results from defects in mRNA and/or protein processing, we quantified the levels of Gigaxonin mRNA in the lymphoblast cell lines of GAN patients and their relatives, as presented in Figures 1 and 2. This analysis revealed that most of the patients present Gigaxonin mRNA levels that are in the range of wild type Gigaxonin mRNAs (Figure 3). Indeed, the fold changes, expressed as the ratio of the levels of the mutated GAN mRNAs to the average of four control mRNAs after normalization with HPRT mRNA levels, were comprised within an interval of 2, that is considered as statistically similar. Nevertheless, four patients displayed mRNA levels that are statistically different form the wild type mRNA levels and all of them carry nonsense or deletion mutations in both copies of their mRNAs (Figure 3A). Thus, the two patients F16.1 and F16.2 displaying both a homozygous R477X mutation present a fold change of 0,27 ± 0,12 and 0,34 ± 0,11, respectively. Affected by another homozygous nonsense mutation (R242X), patient F23 also has mRNA levels below the 2 fold range (0,38 ± 0,12). The patient F18 carrying K338X/Δ6-8 mutations displays the lowest mRNA levels (0,04 ± 0,01). Noteworthy, when present in only on allele, nonsense (C393X for patient F11) or deletion (Δ3-11 for patient F25) mutation does not affect the overall stability of Gigaxonin mRNA. Interestingly, patient F24 who carries a homozygous deletion encompassing exons 10–11 exhibits normal Gigaxonin mRNA level (Figure 3B). Thus, we conclude that nonsense mediated mRNA decay is one of the disease mechanisms leading to decreased abundance of few GAN linked Gigaxonin but that another mechanism is implicated.

### Structural homology modeling of GAN-linked mutations

To date there is no structure of a full-length BTB-BACK-KELCH protein, and accordingly we could not produce the full-length Gigaxonin protein. Nonetheless, structures exist for the BTB-BACK domain of Gigaxonin, for complexes between other BTB-BACK domains and the N-terminal domain of Cul3, and for KELCH domains either alone or, in the case of Keap1, in complex with ubiquitination substrates [17-20,23]. These structures were used as the basis for homology modeling, to examine potential effects of GAN disease mutations.

**Figure 4:**
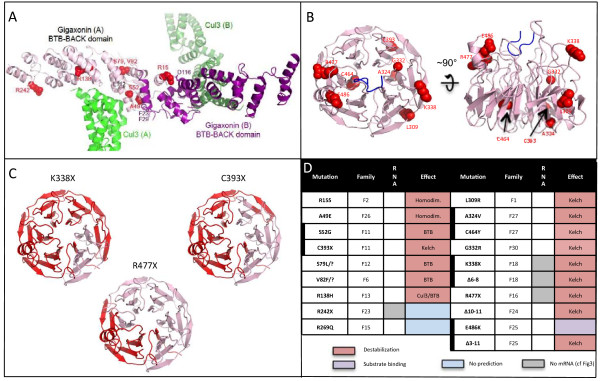
**Structural modeling of Gigaxonin and predicted destabilization due to mutations. A** Structural model of the homodimer BTB-BACK domain of Gigaxonin (purple A, B), in complex with with Cul3 (green A, B). Patient mutations lying in this domain are represented in red. **B** Representation of the top and side views of a structural model for the 6-bladed β-propeller KELCH domain of Gigaxonin. Mutations found in patients are represented in red. **C** Top view of the structural model for the KELCH domain of Gigaxonin, with regions deleted by the indicated truncation mutants shown in red. **D** Summary of the effects predicted from the modelization of Gigaxonin for all patients included in the study. Heterozygous mutations are represented by a thick vertical bar. Most of the mutations are predicted to destabilize Gigaxonin (red), whereas one of them would impair substrate binding (purple). The effect of two mutations could not be determined by the 3D model (blue).

The existing structures of the BTB-BACK domain of Gigaxonin and its docking to Cul3 allowed us to generate hypotheses as to the potential effects of mutations (Figure 4A, D). In structures of the BTB domain from Gigaxonin, amino acid S79 caps the N-terminus of a helix from the BTB domain, S52 and V82 form contacts internal to the BTB domain, whereas R15 and A49 map to the homodimerization interface. R138 is buried between the BACK domain helices that form the Cul3 binding site. Thus, mutation of any of these residues might perturb the folding of Gigaxonin, either through altering packing of the monomer or of the dimer. Mutation of R138 may also directly impact Cul3 binding. Whereas truncation of Gigaxonin at position R242 is expected to produce an intact BTB-BACK fragment, we showed that the decreased abundance of Gigaxonin is caused by activation of nonsense mediated RNA decay in patient F23 (Figures 3, 4A, D).

The modeling suggested that L309, A324, G332, and C464 are all buried between blades of the 6-bladed propeller KELCH domain (Figure 4B, D). This could explain how missense mutations in these positions would destabilize the structure of the KELCH domain. Furthermore, all nonsense or mutations of deletion in Gigaxonin are likely severely destabilizing the structure, by improper folding of the β-propeller that constitutes the KELCH domain (Figure 4C). Non-targeted by nonsense mediated mRNA decay, the destabilization of truncated Gigaxonin is particularly relevant for the mutation C393X, deletions exons3-11 and exon10-11. Whereas all mutations reported earlier are expected to destabilize Gigaxonin by interfering with its homodimerization, its interaction with Cul3 or by impairing the proper folding of either the BTB or the KELCH domain, the mechanism of instability of Gigaxonin mutated at residue E486 may differ. Although the modeling of loops is less accurate due to variations in this region in KELCH domain structures [18], this residue may be located near the upper face of the propeller, and thus could impact protein-protein interactions of the propeller. Notably, the corresponding surface of another BTB-KELCH protein, Keap1, interacts with ubiquitin ligase substrates through this surface. Thus, one could hypothesize that impairing substrate binding may lead to Gigaxonin instability or that the unstable Gigaxonin on the other allele may destabilize the heterodimer E486K/Δ3-11. The R269Q mutation lies outside all regions modeled and its effect is therefore challenging to predict.

### As predicted by Gigaxonin modelization, GAN-linked mutations exhibit shorter protein half-lives

**Figure 5:**
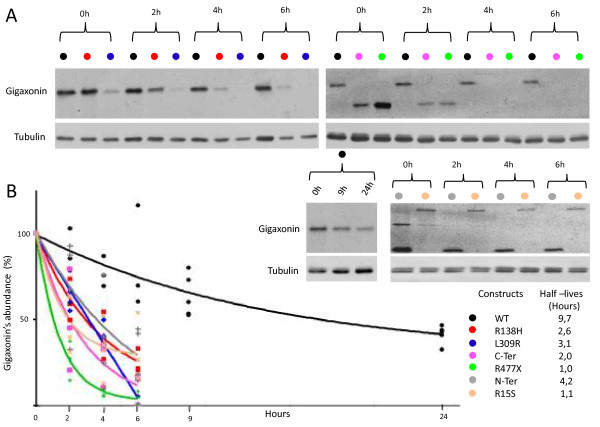
**GAN-linked Gigaxonins exhibit shorter half-lives. A** Representative autoradiograms and immunoblots of the pulse chase assay for wild type, mutant Gigaxonins, and the BTB (N-ter) and KELCH (C-ter) domains. At different times (2, 4, 6, 9, 24 h) after the beginning of the chase (0 h), Gigaxonin was immunoprecipitated and processed for autoradiography (top panel). The signals were normalized to the tubulin immunoblotting of the supernatent fractions of the IPs and plotted at 100% for the time point 0 h. **B** Half-lives of Gigaxonins. Each of the 3–4 experiments realized per construction is plotted on the graph, to define the corresponding curve.

To find out if the predicted instability of mutant Gigaxonins could account for the decreased abundance of Gigaxonin in patients, we determined the half-lives of wild type and mutated Gigaxonin. Extremely challenging to assess in patient’s cells due to the very low abundance of Gigaxonin [8], we combined an overexpressing system in Cos cells and a short-term incubation with ^35^S-methionine/cysteine to radiolabel newly synthetized proteins and to follow their stability over time (Figure 5). To cover the different mechanisms of Gigaxonin instability predicted by the 3D modelization, we selected patient’s mutations affecting the homodimerization domain (R15S), the BTB folding and Cul3 binding (R138H), the folding of the KELCH domain due to single missense mutation (L309R) or massive truncation (R477X). This analysis revealed a great instability of all the mutants tested. Indeed, whereas the estimated half-live of the wild type Gigaxonin is ≈ 10 hours, mutants exhibit half-lives ranging from 1 to 3,1 hours, representing a 3,1-8,8 fold destabilization. Accordingly to the 3D model, Gigaxonin is formed by two distinct folding structures, the BTB and the KELCH domains that are linked together by the BACK domain. To determine whether each domain may affect the stability of the full-length protein or in the contrary whether one folding unit is sufficient to promote stability, we determined the half-lives of both the BTB-BACK and the BACK-KELCH domains, respectively N-ter (corresponding to the R242X mutations in F23) and C-ter (Figure 5). This analysis showed that both fragments of Gigaxonin are less stable than the full protein, with a 2 and a 4,2 hours half-lives for the KELCH and the BTB domain, respectively.

Thus, altogether, the measurement of the stability of its mutant forms provide evidence that GAN mutations confer instability of Gigaxonin in patients, and this notion is corroborated by the homology modeling of mutant locations.

## Discussion

We previously identified Gigaxonin as the defective protein in Giant Axonal Neuropathy [1]. Developing the first molecular diagnostic test for GAN by systematic sequencing of the 11 exons of the *GAN* gene, we identified 23 distinct mutations all along the gene in 22 unrelated families of various geographic origins [1,15,24]. This diagnostic method, complementing the clinical examination of patients is being used worldwide by many groups and proved to be successful in identifying most of the mutations, *i.e.* point mutations or small insertions/deletions in the coding sequence as well as splice mutations near the exons-introns junctions. Nonetheless, this method revealed its limitations in identifying potential non-coding mutations (promotor, intron) or large deletions, as revealed by our inability to detect the heterozygous mutations in patients F6 and F12 [1] and the need to use CGH array for large deletion (patient F25 [16]). Hence, we develop and validate in this study a new diagnostic method specific for GAN, based on the immunodetection of Gigaxonin, that we prove to be very valuable in discriminating GAN from clinically related neuropathies.

We showed in this study that regardless of the type, the position of the mutations or the severity of the disease, all mutated Gigaxonins (as carried by both parental alleles) display a drastic reduction in their abundance, reaching 85,8% in average of the normal level. The effect was so impressive that we further assessed whether the method may be useful for diagnostic purpose. Indeed, the first phase of the disease progression of typical severe forms of GAN, as well as some GAN atypical mild forms -with no involvement of the central nervous system [25,26]- presents a clinical picture closely resembling other frequent peripheral neuropathies such as CMT2. In addition, giant axons filled with aggregated NFs, initially constituting an histopathological hallmark for GAN can also been found in CMT2E and CMT4C patients [4,5]. Thus, we collected seven new patients with a clinical presentation and nerve biopsy suggestive of the severe or milder form of GAN. The high diagnostic value of our new test was validated as univocally, all patients displaying a dramatic reduced level of Gigaxonin were further confirmed by the identification of the corresponding genetic alterations in the *GAN* gene. Combining systematic sequencing to CGH analysis, we identified all *GAN* mutations in 5 unrelated patients, encompassing missense, nonsense mutations and a large deletion. Interestingly, our test was able to suspect heterozygosity in a carrier (sister S1 of patient F24) that was further confirmed by the identification of the large *GAN* deletion on one allele. Noteworthy, patients suspected to bear a mild (F29) but also a severe (F28) form of GAN were excluded by our new test and may be tested for candidate genes in autosomal recessive forms of CMT2 or exome sequencing. Altogether, our study reveals that the clinical evaluation of patients and the histological examination are indeed important but not sufficient to diagnose GAN and differentiate this entity from other frequent peripheral neuropathies. Thus, it is conceivable that the prevalence of GAN is under-evaluated, and that our test will be useful in identifying GAN among related CMTs. Currently being adapted on fresh blood samples, our methodology will enable the community to identify GAN pathogenic variants from targeted diagnosis or following high-speed sequencing analysis, and this in a cost-less and fast manner.

Overcoming the limitation of gene sequencing, the determination of Gigaxonin abundance has proven to be a very important diagnostic tool -specific, reliable and robust- in all GAN families tested so far. Nevertheless, one has to be cautious as some mutations may confer loss of function without necessarily conferring transcript and protein instability. Thus, we propose to determine Gigaxonin abundance prior but in concert with the systematic screening for GAN mutations/deletions in patients, to define in the future the confidence of our methodology as a sufficient diagnostic test for GAN.

We previously determined that Gigaxonin is a new BTB-KELCH protein, predominantly and equally expressed throughout the nervous system but at very low level [8,10]. A key question in understanding how GAN-linked recessive mutations in Gigaxonin cause the disease is to determine how the normal protein is structured and regulated and how patient’s mutations alter its properties. In particular, establishing whether Gigaxonin’s functions are truly mediated by a Cul3-E3 ubiquitin ligase activity requires some knowledge on the stability and the 3D structure of the normal protein. We investigated here the stability of this BTB-KELCH protein, modelized its 3D structure and analyzed the effect of disease-associated mutations on Gigaxonin to identify both mRNA and protein instability as disease mechanisms in GAN. The fact that only patients carrying truncated *GAN* gene on both alleles display a down regulation of their mRNA would indicate a compensatory mechanism in patients carrying compound missense and truncated mutations: either a stabilization or a enhanced transcription of the truncated and missense mRNA, respectively.

The homology modeling of normal Gigaxonin allowed us to predict the structural effects of *GAN* mutations. Regardless of the stability of the mRNA levels, the majority (85%) of the Gigaxonin mutations are predicted to map to buried surfaces, which could alter the folding of the BTB or KELCH domains, the homodimerization of the protein, and/or the interaction with the Cul3 subunit of the E3 ligase. The other 15% mutations may impair substrate binding, which may indirectly still confer instability. Indeed, as suggested for other BTB-KELCH proteins, many cullin-E3 ligase adaptors are destabilized by (auto)-ubiquitination in the absence of substrates, possibly to avoid constitutive activation of the E3 ligase [27]. Accordingly to this hypothesis, mutations interfering with substrate binding might activate Gigaxonin (auto) ubiquitination in patients, leading to its degradation. With the aim to confirm instability of mutant Gigaxonin as the key mechanism in GAN, we demonstrated that all mutations tested, as well as isolated BTB or KELCH structural domains, decreased the half-live of the protein by 2 to 9 fold.

## Conclusions

We have not only developed a new powerful method to diagnose GAN, we have also provided the first evidence that disease-associated mutations confer instability of Gigaxonin in the human pathology. Reconstitution of the E3 ligase activity of the Gigaxonin-Cul3 complex, together with the identification of its partners are now essential to unravel the mechanisms controlled by Gigaxonin in sustaining neuron survival and cytoskeleton architecture. This will shed light onto the role(s) of the BTB-KELCH protein Gigaxonin in Giant Axonal Neuropathy and may contribute in the understanding on how mutations in the UPS contribute to neurodegeneration, as exemplified in Parkinson, Spinocerebellar Ataxia, Angelman syndrome and CMT diseases.

## Competing interests

The authors declare that they have no conflict of interest.

## Authors’ contributions

AB performed the majority of the experiments, analyzed the data and contributed to writing of the manuscript. YTA realized and analyzed some experiments, and helped correcting the manuscript. BR performed a systematic sequencing analysis of the *GAN* gene in patients and identified all punctual mutations. BAS generated the structural model for Gigaxonin, provided prediction of Gigaxonin’s mutations and contributed to the writing of the manuscript. DM and HH developed and applied the CHG methodology for GAN patients. TS identified patients exhibiting inherited peripheral neuropathy, provided clinical data and contributed to the writing of the manuscript. NK, CH and BC identified patients and provided clinical data. PB designed the study, analyzed the data and wrote the manuscript. All authors corrected the manuscript. All authors read and approved the final manuscript.

## References

[B1] Bomont P Cavalier L Blondeau F Ben Hamida C Belal S Tazir M Demir E Topaloglu H Korinthenberg R Tuysuz B Landrieu P Hentati F Koenig M The gene encoding gigaxonin, a new member of the cytoskeletal BTB/kelch repeat family, is mutated in giant axonal neuropathy Nat Genet 2000 26 370 374 10.1038/81701 11062483

[B2] Asbury AK Gale MK Cox SC Baringer JR Berg BO Giant axonal neuropathy: a unique case with segmental neurofilamentous masses Acta Neuropathol 1972 20 237 247 10.1007/BF00686905 5044004

[B3] Berg BO Rosenberg SH Asbury AK Giant axonal neuropathy Pediatrics 1972 49 894 899 4339350

[B4] Azzedine H Ravise N Verny C Gabreels-Festen A Lammens M Grid D Vallat JM Durosier G Senderek J Nouioua S Hamadouche T Bouhouche A Guilbot A Stendel C Ruberg M Brice A Birouk N Dubourg O Tazir M LeGuern E Spine deformities in Charcot-Marie-Tooth 4C caused by SH3TC2 gene mutations Neurology 2006 67 602 606 10.1212/01.wnl.0000230225.19797.93 16924012

[B5] Lus G Nelis E Jordanova A Lofgren A Cavallaro T Ammendola A Melone MA Rizzuto N Timmerman V Cotrufo R De Jonghe P Charcot-Marie-Tooth disease with giant axons: a clinicopathological and genetic entity Neurology 2003 61 988 990 10.1212/WNL.61.7.988 14557576

[B6] Prineas JW Ouvrier RA Wright RG Walsh JC McLeod JG Giant axonal neuropathy: a generalized disorder of cytoplasmic microfilament formation J Neuropathol Exp Neurol 1976 35 458 470 10.1097/00005072-197607000-00006 180266

[B7] Bomont P Koenig M Intermediate filament aggregation in fibroblasts of giant axonal neuropathy patients is aggravated in non dividing cells and by microtubule destabilization Hum Mol Genet 2003 12 813 822 10.1093/hmg/ddg092 12668605

[B8] Cleveland DW Yamanaka K Bomont P Gigaxonin controls vimentin organization through a tubulin chaperone-independent pathway Hum Mol Genet 2009 18 1384 1394 10.1093/hmg/ddp044 19168853 PMC2664145

[B9] Dequen F Bomont P Gowing G Cleveland DW Julien JP Modest loss of peripheral axons, muscle atrophy and formation of brain inclusions in mice with targeted deletion of gigaxonin exon 1 J Neurochem 2008 107 253 264 10.1111/j.1471-4159.2008.05601.x 18680552 PMC3657508

[B10] Ganay T Boizot A Burrer R Chauvin JP Bomont P Sensory-motor deficits and neurofilament disorganization in gigaxonin-null mice Mol Neurodegene 2011 6 25 10.1186/1750-1326-6-25 PMC3094382 21486449

[B11] Furukawa M He YJ Borchers C Xiong Y Targeting of protein ubiquitination by BTB-Cullin 3-Roc1 ubiquitin ligases Nat Cell Biol 2003 5 1001 1007 10.1038/ncb1056 14528312

[B12] Pintard L Willis JH Willems A Johnson JL Srayko M Kurz T Glaser S Mains PE Tyers M Bowerman B Peter M The BTB protein MEL-26 is a substrate-specific adaptor of the CUL-3 ubiquitin-ligase Nature 2003 425 311 316 10.1038/nature01959 13679921

[B13] Xu L Wei Y Reboul J Vaglio P Shin TH Vidal M Elledge SJ Harper JW BTB proteins are substrate-specific adaptors in an SCF-like modular ubiquitin ligase containing CUL-3 Nature 2003 425 316 321 10.1038/nature01985 13679922

[B14] Mahammad S Murthy SN Didonna A Grin B Israeli E Perrot R Bomont P Julien JP Kuczmarski E Opal P Goldman RD Giant axonal neuropathy-associated gigaxonin mutations impair intermediate filament protein degradation J Clin Invest 2013 123 1964 1975 10.1172/JCI66387 23585478 PMC3635735

[B15] Bomont P Ioos C Yalcinkaya C Korinthenberg R Vallat JM Assami S Munnich A Chabrol B Kurlemann G Tazir M Koenig M Identification of seven novel mutations in the GAN gene Hum Mutat 2003 21 446 12655563 10.1002/humu.9122

[B16] Buysse K Vergult S Mussche S Ceuterick-de Groote C Speleman F Menten B Lissens W Van Coster R Giant axonal neuropathy caused by compound heterozygosity for a maternally inherited microdeletion and a paternal mutation within the GAN gene Am J Med Genet A 2010 152A 2802 2804 10.1002/ajmg.a.33508 20949505

[B17] Zhuang M Calabrese MF Liu J Waddell MB Nourse A Hammel M Miller DJ Walden H Duda DM Seyedin SN Hoggard T Harper JW White KP Schulman BA Structures of SPOP-substrate complexes: insights into molecular architectures of BTB-Cul3 ubiquitin ligases Mol Cell 2009 36 39 50 10.1016/j.molcel.2009.09.022 19818708 PMC2847577

[B18] Canning P Cooper CD Krojer T Murray JW Pike AC Chaikuad A Keates T Thangaratnarajah C Hojzan V Marsden BD Gileadi O Knapp S von Delft F Bullock AN Structural basis for Cul3 assembly with the BTB-Kelch family of E3 ubiquitin ligases J Biol Chem 2013 288 7803 7814 10.1074/jbc.M112.437996 23349464 PMC3597819

[B19] Errington WJ Khan MQ Bueler SA Rubinstein JL Chakrabartty A Prive GG Adaptor protein self-assembly drives the control of a cullin-RING ubiquitin ligase Structure 2012 20 1141 1153 10.1016/j.str.2012.04.009 22632832

[B20] Lo SC Li X Henzl MT Beamer LJ Hannink M Structure of the Keap1:Nrf2 interface provides mechanistic insight into Nrf2 signaling EMBO J 2006 25 3605 3617 10.1038/sj.emboj.7601243 16888629 PMC1538563

[B21] Padmanabhan B Tong KI Ohta T Nakamura Y Scharlock M Ohtsuji M Kang MI Kobayashi A Yokoyama S Yamamoto M Structural basis for defects of Keap1 activity provoked by its point mutations in lung cancer Mol Cell 2006 21 689 700 10.1016/j.molcel.2006.01.013 16507366

[B22] Roth LA Johnson-Kerner BL Marra JD Lamarca NH Sproule DM The absence of curly hair is associated with a milder phenotype in Giant Axonal Neuropathy Neuromuscul Disord 2014 24 48 55 10.1016/j.nmd.2013.06.007 23890932

[B23] Tong KI Padmanabhan B Kobayashi A Shang C Hirotsu Y Yokoyama S Yamamoto M Different electrostatic potentials define ETGE and DLG motifs as hinge and latch in oxidative stress response Mol Cell Biol 2007 27 7511 7521 10.1128/MCB.00753-07 17785452 PMC2169061

[B24] Demir E Bomont P Erdem S Cavalier L Demirci M Kose G Muftuoglu S Cakar AN Tan E Aysun S Topcu M Guicheney P Koenig M Topaloglu H Giant axonal neuropathy: clinical and genetic study in six cases J Neurol Neurosurg Psychiatry 2005 76 825 832 10.1136/jnnp.2003.035162 15897506 PMC1739689

[B25] Ben Hamida M Hentati F Ben Hamida C Giant axonal neuropathy with inherited multisystem degeneration in a Tunisian kindred Neurology 1990 40 245 250 10.1212/WNL.40.2.245 2153943

[B26] Zemmouri R Azzedine H Assami S Kitouni N Vallat JM Maisonobe T Hamadouche T Kessaci M Mansouri B Le Guern E Grid D Tazir M Charcot-Marie-Tooth 2-like presentation of an Algerian family with giant axonal neuropathy Neuromuscul Disord 2000 10 592 598 10.1016/S0960-8966(00)00141-3 11053687

[B27] Petroski MD Deshaies RJ Function and regulation of cullin-RING ubiquitin ligases Nat Rev Mol Cell Biol 2005 6 9 20 10.1038/nrm1547 15688063

